# Dynamic remodeling of microglial responses in Alzheimer’s disease: heterogeneous states, spatiotemporal progression, and immunometabolic dysregulation

**DOI:** 10.3389/fcell.2026.1929282

**Published:** 2026-10-13

**Authors:** Simin Han, Shanshan Wang, Jingjing Liu, Xulin Liu, Qinglu Wang, Chang Meng

**Affiliations:** 1 School of Management, Qilu Medical University, Zibo, Shandong, China; 2 College of Sport and Health, Shandong Sport University, Jinan, China; 3 Department of Neurology, Zibo 148 Hospital, Zibo, Shandong, China

**Keywords:** disease-associated microglia, immunometabolic reprogramming, microglial heterogeneity, precision immunotherapy, spatiotemporal dynamics

## Abstract

Alzheimer’s disease (AD) is characterized by progressive neuroinflammation involving microglia whose functional responses extend far beyond the classical M1/M2 framework and form a dynamically heterogeneous continuum. Here, we review recurrent disease-associated microglia (DAM) states and transcriptional programs, including DAM-associated, interferon-responsive, cytokine-responsive, proliferative, antigen-presentation-associated, and lipid-associated features, emphasizing that these labels do not necessarily represent fixed or mutually exclusive cellular subtypes. Across AD progression, the relative engagement and functional consequences of these programs vary according to Aβ and tau burden, local microenvironment, genetic background, metabolic status, and disease stage. DAM-associated programs may support phagocytosis and plaque containment in some contexts, whereas persistent inflammatory and metabolic stress can increase lipid dysregulation, mitochondrial dysfunction, impaired phagocytic competence, and inflammatory signaling in subsets of microglia. Microglia also exert bidirectional effects on tau pathology. Core AD risk pathways, including TREM2 and APOE, together with immunometabolic remodeling, shape microglial plasticity in a context-dependent manner. We discuss the implications of this dynamic framework for stage- and state-aware therapeutic strategies and highlight the need to integrate single-cell, spatial, and multi-omic approaches to resolve microglial trajectories and functional states in human AD.

## 1 Introduction

Alzheimer’s disease (AD), the most common cause of dementia, represents a major and growing public health burden (Alzheimer’s Association, 2024). The typical pathological changes in AD include amyloid plaques formed by the deposition of β-amyloid (Aβ) (Glenner and Wong, 1984; Masters et al., 1985), neurofibrillary tangles formed by the aggregation of hyperphosphorylated tau protein (Brion et al., 1985; Kosik et al., 1986) and neuroinflammation, among others (Saiyisan et al., 2026). These pathological processes collectively lead to irreversible memory decline and cognitive impairment in patients. Among the various pathological mechanisms, neuroinflammation is an important component of AD pathogenesis. Microglia represent major cellular contributors to this process through context-dependent inflammatory and disease-responsive signaling, including the release of pro-inflammatory mediators (Chang et al., 2026; Pan et al., 2026). Recent anti-Aβ monoclonal antibodies have demonstrated that disease-modifying therapy can slow cognitive and functional decline in early symptomatic AD, although these treatments do not halt clinical progression and are associated with important safety and implementation challenges (Sims et al., 2023; van Dyck et al., 2023). These advances therefore do not eliminate the need to identify complementary disease mechanisms and therapeutic targets. In this context, advances in single-cell and spatial-omics technologies have established microglia—the resident immune cells of the brain—as key cellular mediators of AD-related neuroimmune responses (Li and Barres, 2018; Long and Holtzman, 2019; Hong et al., 2016). In early AD, microglia detect Aβ-related pathological cues and engage multiple disease-responsive transcriptional programs, including DAM-associated features. TREM2 contributes importantly to these responses but should not be regarded as a simple switch that uniformly drives a beneficial DAM state. Rather, its functional output depends on ligand and receptor context, downstream signaling configuration, microglial metabolic fitness, and disease stage (Keren-Shaul et al., 2017; Wang et al., 2015; Zhou et al., 2020; Wang et al., 2022; Yeh et al., 2016; Ulland et al., 2017; van Lengerich et al., 2023; Zhao et al., 2022). DAM-associated programs may support phagocytosis and plaque containment in some contexts, but their functional consequences are not uniformly neuroprotective. In the complex and protracted pathological process of Alzheimer’s disease (AD), microglia—as the brain’s principal immune cells—play a role that extends far beyond simply triggering inflammation. In fact, even before disease onset, they assume the critical responsibility of maintaining central nervous system homeostasis. During AD progression, dysregulated microglial responses can contribute to pathological progression (Li and Barres, 2018; Ulland and Colonna, 2018). Under physiological conditions, microglia can be regarded as the “resident surveillants” of the brain (Nimmerjahn et al., 2005). Even in their so-called resting state, they are far from quiescent; their highly ramified processes continuously extend and retract, like antennas scanning the surrounding microenvironment (Li and Barres, 2018). This dynamic surveillance capability primarily relies on the purinergic receptor P2RY12, enabling microglia to sensitively detect subtle abnormal signals—such as leaked ATP, imbalanced neurotransmitters, or nascent pathological protein aggregates (Haynes et al., 2006). A 2020 *in vivo* imaging study showed that microglia rapidly respond to neuronal injury through specialized purinergic junctions, supporting a protective surveillance role (Cserep et al., 2020).

Microglia contribute to the shaping and maintenance of neural circuits through complement-dependent synaptic pruning, in which C1q- and C3-tagged synapses are recognized and cleared by microglia (Paolicelli et al., 2011; Schafer et al., 2012). This process is particularly important during neural circuit development and can also contribute to activity-dependent synaptic remodeling (Paolicelli et al., 2011; Schafer et al., 2012). Notably, this process is not unidirectional.

In the past, our understanding of microglia was long confined to the simplistic M1/M2 dichotomous framework. However, studies over the past 5 years have fundamentally reshaped this view. Today, the field widely recognizes microglia as a highly heterogeneous and dynamically plastic cell population, whose states are better conceptualized as a continuum (Fumagalli et al., 2025; Gosselin et al., 2017). This fundamental shift in understanding has been largely driven by advances in single-cell omics technologies. For instance, single-cell RNA sequencing (scRNA-seq) has revealed multiple DAM states and transcriptional signatures, including the DAM program, in both AD models and human brain tissue (Sheng et al., 2026; Dong et al., 2025). Importantly, transcriptomically defined microglial clusters should not necessarily be interpreted as discrete or lineage-stable biological subtypes. Increasing evidence suggests that commonly described populations, including DAM, interferon-responsive microglia, proliferative microglia, antigen-presentation-associated microglia, and lipid- or inflammatory-associated states, may instead represent partially overlapping and context-dependent transcriptional programs within a dynamic state continuum (Paolicelli et al., 2022; Sun et al., 2023). Individual microglia may simultaneously or sequentially engage multiple programs, while the relative prominence of these programs can vary according to pathological burden, local microenvironment, metabolic status, genetic background, anatomical location, and disease stage. Thus, these commonly used labels are best regarded as operational descriptors of dominant molecular features rather than as definitive evidence of mutually exclusive cellular lineages.

Accordingly, this review first summarizes major DAM states and transcriptional programs, with particular attention to their molecular overlap and functional diversity. We then examine how the relative prominence and functional output of these programs are remodeled across pathological stages, anatomical regions, and local microenvironments, before discussing the genetic, signaling, and immunometabolic mechanisms that shape microglial plasticity and their therapeutic implications.

## 2 Core reactive microglial states and transcriptional programs in AD

To improve conceptual precision, we distinguish here among microglial subtypes, states, and transcriptional programs. We reserve the term “subtype” for a relatively stable and reproducible biological population whose identity is supported by evidence beyond a single transcriptomic clustering solution, such as persistent molecular or functional properties, lineage relationships, or reproducibility across experimental contexts. By contrast, a “state” refers to a context-dependent cellular condition that may change with pathological burden, local microenvironment, disease stage, metabolic status, or signaling history. A “transcriptional program” refers to a coordinated set of genes and associated biological processes that can be engaged to varying degrees across multiple cellular states and may coexist with other programs within the same cell (Fumagalli et al., 2025; Paolicelli et al., 2022; Sun et al., 2023; Mancuso et al., 2024). Accordingly, a transcriptomically defined cluster should not automatically be equated with a stable biological subtype.

Within this framework, terms such as DAM, interferon-responsive microglia, proliferative microglia, antigen-presentation-associated microglia, and inflammatory microglia are used primarily as operational descriptors of dominant molecular or functional features rather than as evidence of rigid and mutually exclusive cellular lineages. Recent single-cell, single-nucleus, and spatial-omics studies indicate that the molecular boundaries among these categories can partially overlap and are strongly shaped by pathological, spatial, genetic, and experimental context (Sun et al., 2023; Mancuso et al., 2024; Lu et al., 2026; Ardura-Fabregat et al., 2025; Mrdjen et al., 2025). Individual microglia may therefore simultaneously engage multiple transcriptional programs or shift between cellular states as the pathological microenvironment changes. Thus, unless stronger evidence supports a stable biological population, these labels are best interpreted as positions or recurrent configurations within a dynamic disease-associated state space rather than as fixed endpoints of differentiation (Fumagalli et al., 2025; Paolicelli et al., 2022; Sun et al., 2023; Lu et al., 2026).

### 2.1 DAM-associated programs across mouse models and human AD

DAM were initially identified by Keren-Shaul et al. using single-cell RNA sequencing in an AD mouse model and were characterized by a disease-responsive transcriptional profile associated with Aβ pathology (Keren-Shaul et al., 2017). The original study proposed a two-phase framework for the acquisition of DAM-associated features, beginning with a TREM2-independent reduction in homeostatic and inhibitory-checkpoint programs, followed by a TREM2-dependent transcriptional response characterized by increased expression of genes involved in lipid metabolism and phagocytosis, including APOE, LPL, and TREM2 (Keren-Shaul et al., 2017; Wang et al., 2015).

Importantly, this two-phase framework should be regarded as a useful model of DAM-associated transcriptional remodeling rather than an obligatory or unidirectional differentiation trajectory. Subsequent single-cell studies in mouse models and human AD have demonstrated substantial context dependence in the expression of DAM-associated signatures, with their magnitude and molecular composition varying according to TREM2 status, pathological environment, species, and genetic background (Zhou et al., 2020; Paolicelli et al., 2022; Sun et al., 2023). More recent analyses of xenografted human microglia exposed to Aβ pathology have further revealed multiple disease-responsive states, including DAM-, interferon-, antigen-presentation-, and cytokine-response-associated programs, together with intermediate transcriptional states, supporting a branched and heterogeneous response landscape rather than a single terminal DAM identity (Mancuso et al., 2024). Accordingly, DAM is considered here as a recurrent disease-associated transcriptional state or program within a broader and dynamic microglial state landscape rather than as a fixed terminal subtype.

Microglial responses in AD can produce both protective and maladaptive functional outcomes, depending on the pathological context and the dominant transcriptional programs engaged. Within DAM-associated states, enhanced phagocytic and lipid-processing programs have been associated with Aβ uptake and plaque containment (Keren-Shaul et al., 2017; Wang et al., 2015). TREM2 signaling can support these responses, whereas AD-associated TREM2 mutations impair phagocytic capacity in human stem cell-derived microglia (Garcia-Reitboeck et al., 2018). In addition, plaque-associated microglia can form barrier-like structures around Aβ deposits, thereby promoting plaque compaction and limiting the local exposure of surrounding neurites to potentially neurotoxic Aβ species (Condello et al., 2015; Yuan et al., 2016). Microglia may also contribute to local tissue homeostasis through context-dependent regulation of synaptic remodeling, neurotrophic support, and responses to tissue injury (Nimmerjahn et al., 2005; Parkhurst et al., 2013; Davalos et al., 2005; Weinhard et al., 2018). Importantly, these protective functions should not be regarded as invariant properties of all DAM-like cells, but rather as context-dependent functional outputs whose magnitude and consequences vary with the local pathological environment and microglial state.

Under sustained pathological stress, subsets of microglia may increasingly engage inflammatory, complement-associated, antigen-presentation, and stress-response programs rather than uniformly converting from a protective DAM state into a fixed neurotoxic phenotype. Persistent inflammatory signaling and excessive complement activation can contribute to synaptic dysfunction and neuronal injury, while microglia may also facilitate the propagation of pathological tau through extracellular vesicle- or exosome-associated mechanisms. Recent human single-cell and spatial-omics studies further indicate that DAM responses vary substantially according to pathological stage, anatomical region, genetic background, and the local Aβ/tau microenvironment (Lu et al., 2026; Mrdjen et al., 2025; Green et al., 2024; Miyoshi et al., 2024; Kosoy et al., 2025). Different immune, antigen-presentation, and stress-associated programs may therefore become enriched, coexist, or recede along disease-associated trajectories rather than following a universal protective-to-neurotoxic sequence. Thus, microglial dysfunction in advanced AD is better conceptualized as context-dependent remodeling of overlapping transcriptional and functional programs rather than as an obligatory conversion of DAM into a single neurotoxic endpoint.

Taken together, the “double-edged sword” role of microglia is better understood as a manifestation of context-dependent functional plasticity rather than a binary distinction between protective and neurotoxic cell populations. Importantly, apparently conflicting reports of beneficial or detrimental DAM-associated functions may reflect differences in pathological burden, disease stage, anatomical region, genetic background, and local microenvironment, as well as differences in the relative engagement of overlapping phagocytic, inflammatory, antigen-presentation, metabolic, and stress-response programs (Lu et al., 2026; Mrdjen et al., 2025; Green et al., 2024; Miyoshi et al., 2024; Kosoy et al., 2025). Thus, DAM-associated responses should be interpreted according to their experimental and pathological context rather than assigned a uniformly protective or detrimental role.

These context-dependent DAM-associated functional outputs and major remodeling tendencies are summarized schematically in Figure 1.

**FIGURE 1:**
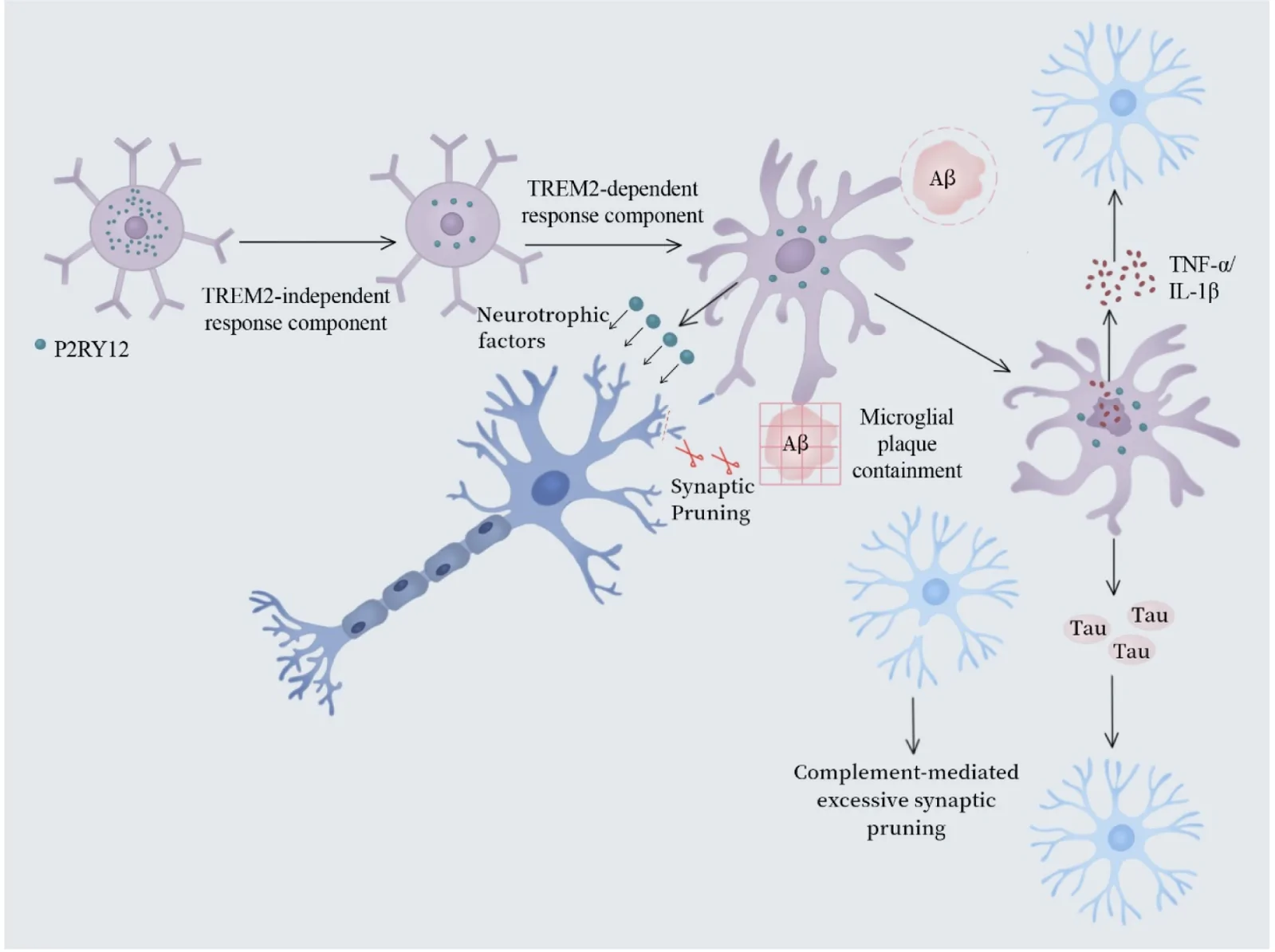
Dynamic Functional States and Major Remodeling Tendencies of Microglia in Alzheimer’s Disease. Schematic representation of major disease-associated microglial remodeling tendencies in Alzheimer’s disease (AD). In response to Aβ-related pathological cues, homeostatic microglia may progressively acquire DAM-associated features through transcriptional changes involving TREM2-independent and TREM2-dependent components. The arrows summarize major experimentally observed tendencies and should not be interpreted as obligatory, unidirectional, or lineage-defined transitions. DAM-associated and other reactive microglial programs may contribute to context-dependent protective functions, including Aβ plaque containment, phagocytic support, modulation of synaptic remodeling, and neurotrophic support. Under sustained pathological stress, inflammatory, complement-associated, and dysfunctional programs may become increasingly prominent in subsets of microglia, contributing to excessive complement-associated synaptic pruning, impaired phagocytic function, and facilitation of tau pathology. These changes represent context-dependent remodeling of overlapping microglial programs rather than an obligatory conversion of DAM into a single neurotoxic endpoint.

### 2.2 Proliferative microglia: a cell-cycle-associated state in AD

Proliferative, or cycling, microglia are more appropriately viewed as microglia engaging a transient cell-cycle-associated transcriptional state rather than as a stable lineage-defined subtype (Paolicelli et al., 2022; Mathys et al., 2024). This state is characterized by increased expression of cell-cycle genes, including MKI67, TOP2A, STMN1, and other mitotic regulators (Mancuso et al., 2024; Mathys et al., 2024). Proliferative microglia have been detected in both experimental AD models and human AD transcriptomic datasets, and recent multiregional single-nucleus profiling of the human AD brain has identified an MKI67-positive cycling microglial program among the major immune-cell expression modules (Mathys et al., 2024). Similarly, xenografted human microglia exposed to Aβ pathology contain a distinct population enriched for proliferation-associated genes such as TOP2A, MKI67, and STMN1 (Mancuso et al., 2024). These findings support the interpretation of proliferation as a dynamic cellular program that can emerge within the broader disease-responsive microglial landscape rather than as evidence of an independent and permanently committed microglial lineage (Paolicelli et al., 2022; Mathys et al., 2024).

In AD-related contexts, proliferative activity may precede, accompany, or overlap with the acquisition of plaque-associated and DAM-like transcriptional features rather than constituting a mandatory precursor-to-DAM differentiation pathway (Ardura-Fabregat et al., 2025; Villacampa et al., 2025). Recent fate-mapping and single-cell analyses in an amyloid mouse model showed that both plaque-associated and non-plaque-associated microglia can exhibit proliferative activity, while non-plaque-associated microglia can subsequently contribute to plaque-associated states after Aβ deposition in a process influenced by local environmental signals such as CSF1 (Ardura-Fabregat et al., 2025). Thus, proliferation may contribute to the local expansion and remodeling of disease-responsive microglial populations, but its functional consequences should not be assumed to be uniformly protective. Indeed, recent work in APP/PS1 mice demonstrated that proliferation-associated transcriptional signatures change with age and Aβ burden and that newly proliferated microglia can display impaired Aβ clearance *in vivo* (Villacampa et al., 2025). Collectively, these findings suggest that proliferative activity represents a context-dependent component of microglial plasticity whose relationship with DAM-associated programs and Aβ handling varies with pathological stage and local microenvironment (Ardura-Fabregat et al., 2025; Villacampa et al., 2025).

Aging also modifies proliferative microglial programs, although the extent to which these changes directly influence AD susceptibility remains uncertain (Tsai et al., 2025).

### 2.3 Interferon-responsive microglia (IRM): a dynamic type I interferon-responsive state

Interferon-responsive microglia (IRM) are more appropriately viewed as microglia engaging a type I interferon (IFN-I)-responsive transcriptional program rather than as a stable lineage-defined subtype. This state is characterized by increased expression of interferon-stimulated genes, including IFIT2, IFIT3, IFITM3, IRF7, and OASL2, and has been identified in AD mouse models and human AD-related datasets (Sala Frigeri et al., 2019; Roy et al., 2022; Roy et al., 2020). More recent single-cell analyses of xenografted human microglia exposed to Aβ pathology have identified IRM alongside DAM-, HLA/antigen-presentation-, cytokine-responsive-, and intermediate states, supporting substantial overlap and heterogeneity among DAM programs (Mancuso et al., 2024). In addition, local immune interactions can influence the prominence and functional consequences of the IFN-responsive program; for example, CD8^+^ T cells promote the accumulation of interferon-activated microglia with myelin-damaging properties in amyloidosis models (Kedia et al., 2024). Persistent or excessive IFN-I signaling can contribute to neuroinflammation, complement-associated synaptic loss, and memory impairment (Roy et al., 2022; Roy et al., 2020). The cGAS-STING pathway represents an important upstream mechanism capable of promoting this response, as Aβ- and tau-associated mitochondrial stress can induce cytosolic DNA accumulation and STING-dependent type I interferon and inflammatory signaling in microglia (Chung et al., 2024). Thus, IRM should not be regarded as an inherently neurotoxic and fixed microglial subtype, but rather as a context-dependent IFN-responsive state whose functional consequences depend on the magnitude and persistence of IFN signaling and on interactions with other disease-associated programs.

### 2.4 Antigen-presentation-associated microglial states

Antigen-presentation-associated microglia are more appropriately viewed as microglia engaging an MHC-II/HLA-enriched transcriptional program rather than as a stable lineage-defined subtype. This state is characterized by increased expression of MHC-II-related genes, including HLA-DRA and CD74, together with genes involved in antigen processing and immune-cell interaction (Sun et al., 2023; Olah et al., 2020). Recent single-cell analyses of xenografted human microglia exposed to Aβ pathology identified an HLA-associated state alongside DAM-, interferon-responsive-, cytokine-responsive-, and intermediate states, with trajectory analyses suggesting partial continuity between DAM- and HLA-associated programs (Mancuso et al., 2024). Experimental amyloidosis studies further indicate that MHC-II-associated microglial features can be shaped by the local immune environment, particularly through interactions with CD8^+^ T cells and interferon signaling (Kedia et al., 2024). Thus, antigen-presentation-associated signatures are better interpreted as context-dependent components of the broader disease-responsive microglial landscape rather than as evidence of a discrete and permanently committed cell population.

The functional significance of antigen-presentation-associated microglial programs in AD appears to be highly context dependent rather than uniformly protective or pathogenic. In amyloidosis models, CD8^+^ T cells promote the accumulation of MHC-II-high microglial states, whereas depletion of CD8^+^ T cells reduces MHC-II-positive microglia; spatial analyses further show increased MHC-II and interferon-responsive signatures in microglia located near T cells (Kedia et al., 2024). Human AD tissue similarly shows close spatial association between CD8^+^ T cells and MHC-II-positive myeloid cells, although spatial proximity alone does not establish a direct antigen-specific interaction (Kedia et al., 2024). Recent human spatial transcriptomic and single-nucleus analyses further identified a transition toward antigen-presentation-enriched microglia programs around the Aβ–tau pathological inflection point (Lu et al., 2026). Importantly, similar late antigen-presentation-associated programs were also observed in cognitively resilient individuals in whom they were partly uncoupled from tau accumulation, indicating that these states are not intrinsically pathogenic (Lu et al., 2026). Spatial proteomic studies likewise reveal substantial regional and microenvironmental variation in human AD microglial states, including variation in HLA-DR expression (Mrdjen et al., 2025). Collectively, these findings suggest that antigen-presentation-associated programs represent dynamic and context-sensitive components of the broader microglial response rather than a simple early-protective/late-pathogenic phenotype.

### 2.5 Cytokine-responsive and inflammatory microglial programs (CRM)

Cytokine-responsive microglia (CRM) are better conceptualized as microglia engaging a cytokine- and chemokine-enriched inflammatory transcriptional program rather than as a fixed inflammatory effector subtype. This program is characterized by increased expression of pro-inflammatory mediators and chemokines, including IL1B, TNF, CCL3, and CCL4 (Sun et al., 2023). Recent single-cell analyses have further identified multiple cytokine-responsive states, including CRM-1, CRM-2, and a transitioning CRM (tCRM) state, alongside DAM-, interferon-responsive-, and HLA-associated states. The presence of intermediate states retaining both homeostatic and CRM-associated features supports the view that cytokine-responsive programs can emerge through partially overlapping and transitional states rather than representing a single discrete cellular identity (Mancuso et al., 2024). Through secretion of chemokines such as CCL3 and CCL4, CRM-associated inflammatory signaling may promote the recruitment of peripheral immune cells, including CD8^+^ T lymphocytes, into the brain parenchyma (Zhang et al., 2025). These T cells, in turn, secrete large amounts of IFN-γ, perforin, and granzymes, which further stimulate microglia to produce more IL-1β, TNF-α, and other factors, thereby exacerbating neuroinflammation.

The expression and functional consequences of inflammatory microglial programs can also be shaped by metabolic dysregulation. For example, loss of MFE-2 and LysoPS–GPR34 signaling illustrate how lipid and metabolic perturbations can reinforce inflammatory programs and impair microglial phagocytic competence (Gao et al., 2025; Zhou et al., 2025). Together, these findings indicate that CRM represents a heterogeneous and context-dependent inflammatory program whose expression and functional consequences vary with pathological and metabolic cues, rather than a single terminal neuroinflammatory subtype.

### 2.6 Human AD microglial states and cross-species considerations

Recent single-cell, single-nucleus, and spatial-omics studies of the human AD brain have revealed extensive microglial heterogeneity and substantial variation in disease-associated transcriptional programs across individuals, brain regions, and pathological contexts (Sun et al., 2023; Mrdjen et al., 2025; Green et al., 2024; Kosoy et al., 2025; Mathys et al., 2024). Rather than representing a set of mutually exclusive human-specific subpopulations, many of these profiles are better interpreted as context-dependent cellular states or transcriptional programs with partially overlapping molecular features. The states discussed below are therefore used as operational descriptors of dominant molecular and functional features rather than as evidence of fixed human-specific microglial lineages.

Lipid-associated microglial states commonly show increased expression of genes involved in lipid sensing, transport, and lysosomal processing, including APOE, GPNMB, LIPA, and TREM2, while the relative prominence of these signatures varies according to genetic background and local pathological context (Green et al., 2024; Haney et al., 2024; Lee et al., 2026). The functional consequences of this metabolic remodeling are context dependent. Enhanced lipid-handling programs may support the processing and clearance of lipid-rich or pathological material (Wang et al., 2015; Lee et al., 2026), whereas excessive or dysregulated lipid storage can promote intracellular lipid-droplet accumulation and become associated with impaired phagocytic function and inflammatory responses (Haney et al., 2024; Marschallinger et al., 2020). These findings indicate that lipid-associated programs can support either adaptive lipid handling or maladaptive lipid accumulation depending on disease context, rather than defining a single uniformly functional or dysfunctional microglial subtype (Green et al., 2024; Haney et al., 2024; Lee et al., 2026).

Autophagy–lysosomal activity is better viewed as a functional program that intersects with other disease-responsive microglial states rather than as a discrete microglial subtype. In AD mouse models, autophagy is enhanced particularly in plaque-associated DAM-like microglia, and disruption of microglial autophagy reduces microglial engagement with amyloid plaques, suppresses DAM-associated responses, and promotes senescence-like dysfunction (Choi et al., 2023). Genetic background can further modify this program. In experimental microglial models, APOE4 reduces autophagic flux and impairs the uptake and clearance of Aβ compared with APOE2-or APOE3-expressing microglia (Bassal et al., 2025). Thus, autophagy-associated features are better interpreted as a context-dependent functional program that can overlap with DAM- and senescence-associated states rather than as a fixed standalone microglial identity.

Stress- and redox-response features are better viewed as context-dependent transcriptional and functional programs that can be engaged across multiple disease-responsive microglial states rather than as a discrete microglial subtype. Human single-nucleus studies have identified microglial profiles enriched for stress-response and redox-related programs, supporting redox regulation as one component of broader microglial heterogeneity in aging and AD (Cain et al., 2023). The functional consequences of these programs are highly context dependent. Experimental studies indicate that excessive oxidative stress can alter microglial function through pathways involving NRF2–KEAP1 and inflammatory signaling, whereas restoration of NRF2 activity can attenuate maladaptive microglial responses (Kang et al., 2024). More recent work has identified an integrated stress response (ISR)-associated microglial program linked to dark-microglial features, toxic lipid secretion, synaptic loss, and tau-related pathology, while inhibition of the ISR mitigated several of these effects in experimental AD models (Flury et al., 2025). Thus, stress- and redox-associated features are likely to overlap with inflammatory, lipid-associated, and other disease-responsive programs rather than defining a single stable microglial identity.

Alterations in sphingolipid and ceramide metabolism are better viewed as metabolic programs that can intersect with other disease-responsive microglial states rather than as a discrete sphingolipid-defined subtype. Human iPSC-derived microglia express key enzymes involved in *de novo* ceramide synthesis and sphingolipid turnover, including SPTLC1/2, members of the CERS family, and ASAH1, indicating a substantial intrinsic capacity for sphingolipid remodeling (McInnis et al., 2024). Experimental studies further indicate that tau-associated pathological stress can remodel microglial sphingolipid metabolism. In microglia exposed to tau-enriched brain extracts, sphingolipids accumulate together with inflammatory and lipid-droplet-associated changes, while disruption of lipid homeostasis is accompanied by impaired tau phagocytosis (Le et al., 2023). Human CSF lipidomic studies further link specific sphingolipid species to AD pathology. Higher levels of Cer(d38:4) were associated with Aβ42 pathological positivity, whereas higher sphingomyelin SM (30:1) levels were associated with pathological phosphorylated-tau levels (Dakterzada et al., 2024). Importantly, these CSF lipidomic associations are not microglia-specific and should therefore be interpreted as evidence of broader sphingolipid dysregulation in AD rather than as direct markers of a distinct microglial state.

Representative metabolic and stress-response programs discussed above, together with their pathological contexts, functional implications, and major evidence sources, are summarized in Table 1.

**TABLE 1:** Representative metabolic and stress-response programs associated with microglial remodeling in Alzheimer’s disease.

Microglial program/Feature	Representative genes/Pathways	AD-related context	Context-dependent functional implications	Evidence/Model context	References
Lipid metabolism/processing program	*APOE, GPNMB, LIPA, TREM2, ACSL1*	Lipid-associated programs vary with Aβ pathology, APOE genotype, and local pathological context	May support lipid handling and pathological-material processing, whereas dysregulated lipid storage is associated with lipid-droplet accumulation, impaired phagocytosis, and inflammatory responses	Human postmortem snRNA-seq; human iPSC-derived microglia; chimeric and mouse models	(Green et al., 2024; Haney et al., 2024; Marschallinger et al., 2020; Claes et al., 2021)
Autophagy–lysosomal program	MAP1LC3B, SQSTM1, BECN1 and related autophagy–lysosomal machinery	Autophagy–lysosomal activity is particularly engaged in plaque-associated and DAM-like microglia and can be modified by pathological burden and genetic background, including APOE genotype	Supports plaque engagement and protein/lipid processing, whereas autophagy impairment may promote senescence-like dysfunction and reduced Aβ handling	AD mouse models; experimental microglial models	(Choi et al., 2023; Bassal et al., 2025)
Stress/redox-response program	HMOX1, NFE2L2/NRF2-related antioxidant pathways, SOD2, integrated stress-response pathways	Stress/redox-related profiles occur within broader aging- and AD-associated microglial heterogeneity	May support adaptive stress responses, whereas persistent oxidative or integrated stress signaling can overlap with inflammatory and lipid-associated dysfunction	Human snRNA-seq; human experimental models; AD/tauopathy mouse models	(Cain et al., 2023; Kang et al., 2024; Flury et al., 2025)
Sphingolipid/ceramide metabolic program	SPTLC1/2, CERS family, ASAH1 and related sphingolipid-turnover pathways	Tau-associated stress alters microglial sphingolipid metabolism; specific ceramide and sphingomyelin species are associated with human AD biomarkers	Dysregulated sphingolipid metabolism can accompany inflammation, lipid-droplet accumulation, and impaired tau phagocytosis; the functional outcome depends on cellular and pathological context	Human iPSC-derived microglia; mouse tauopathy models; human CSF lipidomics	(McInnis et al., 2024; Le et al., 2023; Dakterzada et al., 2024)
Mitochondrial and glycolytic remodeling	OXPHOS, glycolysis, mTOR signaling, mitochondrial fission, mitophagy	AD-relevant microglial responses involve altered mitochondrial energetic capacity and metabolic flexibility; glycolytic shifts have also been observed in general inflammatory and toxicological activation models	Preserved mitochondrial function supports energetic fitness and disease-responsive microglial activity, whereas mitochondrial dysfunction may impair phagocytosis and promote inflammatory signaling; a universal OXPHOS-to-glycolysis transition in AD is not established	AD mouse models; human iPSC-derived microglia; general inflammatory and toxicological models	(Ulland et al., 2017; van Lengerich et al., 2023; Wu et al., 2020; Nair et al., 2019; Lauro and Limatola, 2020)
Cholesterol trafficking and fatty-acid oxidation	TREM2, cholesterol transport/efflux, cholesteryl ester metabolism, CPT1-dependent FAO, acyl-carnitines	Chronic phagocytic challenge and amyloid pathology can disrupt cholesterol clearance and mitochondrial lipid utilization, particularly when TREM2 signaling is impaired	Impaired cholesterol processing promotes cholesteryl-ester and lipid-droplet accumulation, whereas enhanced TREM2 signaling can improve cholesterol homeostasis, FAO, and respiratory capacity	TREM2-deficient microglia; human iPSC-derived microglia; APP-transgenic mouse models	(van Lengerich et al., 2023; Nugent et al., 2020; Feiten et al., 2026)
Glutamine metabolism and NAD+-dependent signaling	Glutaminolysis, AMPK–mTORC1, NLRP3, mitophagy, NAD+, SIRT1, cGAS–STING	Glutamine utilization and NAD+ availability modulate inflammatory and mitochondrial pathways in AD-related and aging/tauopathy models	Altered glutamine metabolism can reinforce NLRP3-associated inflammation, whereas glutaminase inhibition, NAD+ restoration, and SIRT1-dependent signaling can enhance mitophagy and attenuate inflammatory responses; relevance to specific human AD microglial states remains incompletely defined	APP/PS1 mice; activated microglial cultures; aging and tauopathy models	(Zhang et al., 2024; Hou et al., 2021; Cho et al., 2015)

DAM-like transcriptional programs are robust and recurrent features of microglial responses in many mouse models of amyloid pathology (Paolicelli et al., 2022; Ardura-Fabregat et al., 2025), whereas human *postmortem* brain studies reveal a substantially more heterogeneous repertoire of disease-responsive states, including coexisting lipid-metabolic, inflammatory, interferon-responsive, antigen-presentation, proliferative, and stress-associated programs (Sun et al., 2023; Lu et al., 2026; Mrdjen et al., 2025; Green et al., 2024; Miyoshi et al., 2024; Kosoy et al., 2025; Mathys et al., 2024). Experimental mouse models provide important advantages because genotype, age, pathological burden, and sampling time can be controlled and causal perturbations can be tested. However, most models reproduce only selected components of AD pathology and cannot fully recapitulate the combined effects of advanced age, mixed Aβ/tau pathology, comorbidities, and human genetic diversity. Xenografted human microglia partially bridge this gap by allowing human cells to be examined under experimentally controlled AD-related pathological conditions, although their responses remain embedded within a non-human host microenvironment (Mancuso et al., 2024). Thus, differences between mouse and human datasets are better interpreted as differences in the relative weighting and combination of shared and species-enriched disease-responsive programs rather than as evidence for a single conserved sequence of microglial activation (Fumagalli et al., 2025; Paolicelli et al., 2022; Sun et al., 2023; Mancuso et al., 2024; Haney et al., 2024; Jung et al., 2025).

Conversely, human *postmortem* studies provide direct access to disease-relevant human tissue but introduce important limitations for the interpretation of microglial states. *Postmortem* datasets are inherently cross-sectional and may include substantial heterogeneity in disease stage, brain region, genetic background, comorbidities, agonal conditions, *postmortem* interval, tissue preservation, and RNA quality. These variables can influence stress-responsive and immune-related transcriptional profiles and complicate the distinction between *in vivo* disease biology and pre-analytical effects (Fumagalli et al., 2025; Gosselin et al., 2017; Sun et al., 2023; Green et al., 2024; Miyoshi et al., 2024; Kosoy et al., 2025; Mathys et al., 2024). The sequencing platform introduces an additional source of variation. scRNA-seq captures whole-cell transcriptomes but generally requires viable tissue dissociation, which can perturb the transcriptional profile of environmentally sensitive microglia. In contrast, snRNA-seq is readily applicable to frozen *postmortem* tissue and reduces some constraints associated with whole-cell dissociation, but it samples nuclear rather than whole-cell RNA and may incompletely capture cytoplasmic or activation-associated transcripts (Fumagalli et al., 2025; Gosselin et al., 2017; Miyoshi et al., 2024; Kosoy et al., 2025; Mathys et al., 2024; Olah et al., 2020). Therefore, apparent differences in microglial state abundance or marker expression across studies may reflect both biological heterogeneity and methodological sampling.

Cell-state annotation represents a further source of inconsistency across datasets. Differences in clustering resolution, marker-gene selection, batch-correction and integration strategies, reference mapping, brain-region sampling, and nomenclature can result in similar biological programs receiving different labels, while similarly named states may not necessarily represent equivalent transcriptional configurations (Fumagalli et al., 2025; Paolicelli et al., 2022; Sun et al., 2023; Lu et al., 2026; Mrdjen et al., 2025; Green et al., 2024; Miyoshi et al., 2024; Kosoy et al., 2025; Mathys et al., 2024). Cross-dataset comparisons should therefore prioritize conserved gene modules, pathway activity, spatial context, and functional validation rather than relying solely on one-to-one matching of cluster names. These considerations raise a central interpretive question: do the diverse microglial profiles identified in human AD represent stable cellular populations, or context-dependent combinations of partially overlapping programs within a shared state space? Current evidence favors the latter interpretation, although the relative contributions of species, anatomical region, pathological burden, genetic background, and analytical approach remain incompletely resolved (Fumagalli et al., 2025; Paolicelli et al., 2022; Sun et al., 2023; Green et al., 2024). Longitudinal and multi-region human studies will be important for determining whether particular combinations of microglial programs track cognitive trajectories or therapeutic responses (Gazestani et al., 2023). These unresolved methodological and translational challenges are discussed further in Section 5.2.

## 3 Spatiotemporal remodeling of microglial states during AD progression

The disease-responsive microglial states and transcriptional programs described above are not static; their relative abundance, molecular composition, and functional outputs vary across anatomical locations and pathological stages. In the following section, we examine how the relative engagement of these programs changes along Aβ and tau pathological continua and across regional microenvironments (Figure 2). Importantly, the stage-based framework used here summarizes major disease-associated tendencies rather than implying obligatory or unidirectional lineage transitions between discrete microglial populations. The relative prominence and inferred relationships among microglial states should also be interpreted in light of disease model, species, brain region, sex, age, pathological stage, genetic background, and analytical methodology (Paolicelli et al., 2022; Sun et al., 2023; Lu et al., 2026; Mrdjen et al., 2025; Green et al., 2024; Mathys et al., 2024; Sala Frigeri et al., 2019). Accordingly, the early-, intermediate-, and advanced-stage organization used below is intended as a heuristic framework for summarizing recurrent tendencies rather than a universally established trajectory experienced by all microglia.

**FIGURE 2:**
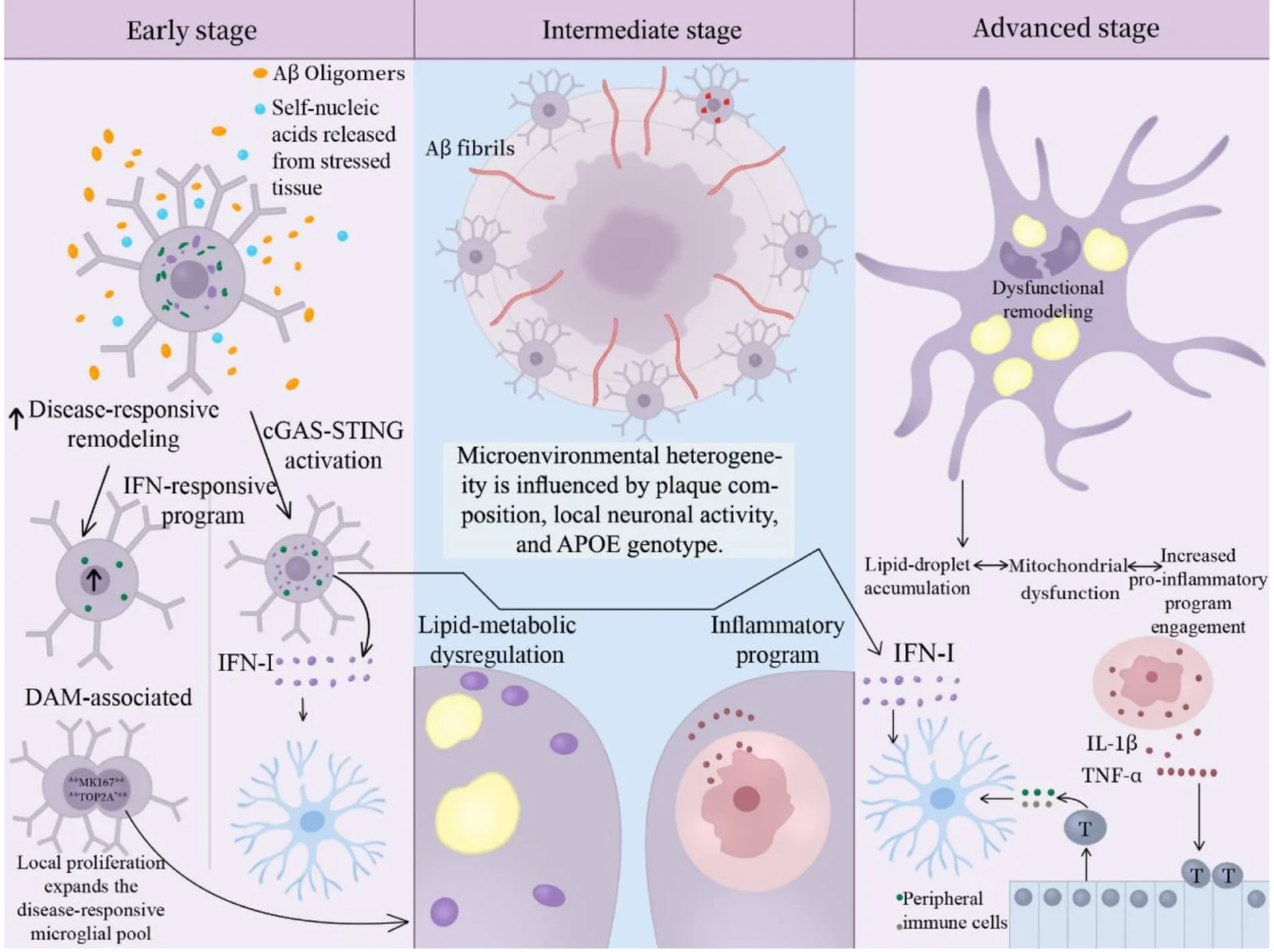
Stage-Associated Remodeling of Microglial States During Alzheimer’s Disease Progression. Early stage: During early amyloid accumulation, Aβ-related pathological cues may promote progressive loss of homeostatic features and increased engagement of DAM-associated transcriptional programs through TREM2-independent and TREM2-dependent components. In parallel, cytosolic nucleic-acid signals can activate cGAS–STING signaling and enhance type I interferon-responsive programs. Local proliferation may further expand disease-responsive microglial populations. These programs may coexist or overlap within individual microglia rather than representing mutually exclusive or obligatory sequential states. Intermediate stage: As plaque burden increases, plaque-associated microglia may show enhanced DAM-associated, phagocytic, and plaque-containment features. At the same time, microenvironmental heterogeneity—including plaque composition, local neuronal activity, and APOE genotype—can differentially shape lipid-metabolic and inflammatory programs. These features may occur in different combinations and to different degrees rather than representing an obligatory divergence of DAM into discrete downstream subtypes. Advanced stage: During advanced AD pathology, sustained amyloid burden, inflammatory signaling, and metabolic stress may increase the prominence of lipid-droplet-associated dysfunction, mitochondrial impairment, and interferon- or cytokine-responsive programs in subsets of microglia. These changes can be accompanied by reduced phagocytic competence and enhanced inflammatory signaling. Rather than representing a uniform transition from DAM to a terminal neurotoxic phenotype, the late-stage response is depicted as a heterogeneous and context-dependent redistribution of partially overlapping microglial functional programs. Arrows indicate predominant and potentially reversible shifts in the relative engagement of microglial programs and do not represent obligatory, unidirectional, or lineage-defined cell-state transitions.

### 3.1 Microglial state remodeling along the Aβ pathological continuum

#### 3.1.1 Early stage: Aβ oligomers and the Initiation of microglial activation

During early amyloid accumulation, soluble Aβ species and other tissue-derived danger signals alter the local microenvironment. Surveillant homeostatic microglia can sense these changes through pattern-recognition and purinergic signaling pathways, including Toll-like receptors and P2RY12-associated signaling (Haynes et al., 2006). Early disease-responsive remodeling can include reduced expression of homeostatic markers such as P2RY12. Within the original two-step DAM framework, this early reduction in homeostatic features was described as a TREM2-independent component of disease-responsive transcriptional remodeling (Keren-Shaul et al., 2017). Microglia may progressively reduce homeostatic programs while increasing transcriptional and functional features associated with chemotaxis, phagocytosis, and responses to tissue stress. This early response is heterogeneous, and subsets of microglia may begin to engage DAM-associated transcriptional features while retaining or simultaneously engaging other disease-responsive programs, rather than following a uniform and irreversible trajectory toward a terminal DAM identity (Keren-Shaul et al., 2017; Mancuso et al., 2024).

In parallel, Aβ-related stress and cytosolic nucleic-acid signals can activate pathways such as cGAS–STING and promote type I interferon-responsive transcriptional programs (Roy et al., 2022; Roy et al., 2020; Chung et al., 2024). These IFN-responsive features may coexist with or emerge alongside DAM-associated and other disease-responsive programs rather than defining a completely separate cellular trajectory (Mancuso et al., 2024). Major early disease-responsive programs and their potential overlap are summarized schematically in Figure 2. In experimental amyloid contexts, early engagement of IFN-I-responsive programs can enhance inflammatory and complement-associated signaling, potentially contributing to excessive synaptic elimination and impaired neural connectivity (Hong et al., 2016; Roy et al., 2022; Roy et al., 2020).

Concurrent with these transcriptional responses, local microglial proliferation can contribute to the expansion of disease-responsive microglial populations around amyloid pathology. A proliferative or cell-cycle-associated microglial state, characterized by increased expression of genes such as MKI67 and TOP2A, has been detected in experimental AD models and human AD transcriptomic datasets (Mathys et al., 2024; Mathys et al., 2019). Local proliferation may contribute to the expansion of plaque-associated microglial populations, although proliferative activity is not restricted to plaque-associated cells and its functional consequences vary with spatial and pathological context. Proliferating microglia may subsequently acquire or overlap with plaque-associated and DAM-like transcriptional features, while recent fate-mapping studies indicate that both plaque-associated and non-plaque-associated microglia can proliferate and dynamically contribute to plaque-associated states (Ardura-Fabregat et al., 2025). Age also modifies proliferative and disease-responsive microglial programs, although the extent to which altered proliferative capacity directly contributes to age-related AD susceptibility remains uncertain (Sala Frigeri et al., 2019).

#### 3.1.2 Intermediate stage: plaque formation and functional specialization

As Aβ burden increases and insoluble amyloid plaques become more prominent, plaque-associated microglia show enhanced disease-responsive transcriptional remodeling. TREM2-dependent signaling contributes to the enrichment of DAM-associated features, including increased expression of lipid metabolism genes such as *Apoe* and *Lpl* and phagocytosis-related genes such as *Cst7* and *Clec7a* (Keren-Shaul et al., 2017; Wang et al., 2015). However, this process should not be interpreted as uniform polarization into a discrete DAM subtype. Rather, individual microglia may express DAM-associated features to different degrees while simultaneously engaging other lipid-processing, inflammatory, interferon-responsive, or proliferative programs according to their local pathological context.

At this stage, plaque-associated microglia may engage phagocytic and plaque-containment programs (Keren-Shaul et al., 2017; Wang et al., 2015; Condello et al., 2015); however, the magnitude and functional consequences of these responses vary across local pathological environments. Spatial transcriptomic studies further reveal substantial heterogeneity among plaque-associated microglia (Green et al., 2024), with lipid-metabolic, inflammatory, and other disease-responsive programs differentially enriched according to plaque composition, local neuronal activity, and genetic background, including APOE genotype (Sun et al., 2023; Green et al., 2024; Haney et al., 2024). Thus, the intermediate stage is better viewed as a context-dependent redistribution and overlap of disease-responsive programs rather than an obligatory divergence of DAM into discrete downstream subtypes (Figure 2).

#### 3.1.3 Advanced stage: chronic pathological Load and context-dependent microglial dysfunction

With advancing AD pathology, persistent amyloid burden, inflammatory signaling, and metabolic stress can reshape the relative engagement of microglial functional programs. Subsets of microglia may exhibit impaired phagocytic competence, altered lipid handling, mitochondrial dysfunction, and enhanced inflammatory signaling, although these features occur in variable combinations rather than defining a uniform late-stage phenotype (Haney et al., 2024; Marschallinger et al., 2020). Importantly, these changes should not be interpreted as an obligatory conversion of DAM into a single dysfunctional or pro-inflammatory endpoint. Instead, advanced AD is characterized by a context-dependent redistribution of partially overlapping DAM-associated, lipid-metabolic, inflammatory, and stress-response programs, whose relative prominence is influenced by local pathology and genetic background.

Persistent inflammatory cues, including type I interferon and cytokine signaling, can increase the relative prominence of interferon- and cytokine-responsive transcriptional programs in subsets of microglia. Sustained type I interferon signaling has been associated with enhanced neuroinflammation, synaptic loss, and memory impairment in experimental AD models (Roy et al., 2022; Roy et al., 2020). Cytokine-responsive and other inflammatory microglial programs may likewise contribute to elevated production of inflammatory mediators and broader neuroimmune crosstalk. These responses can coexist with DAM-associated or lipid-metabolic features rather than defining an obligatory terminal “neurotoxic” microglial identity.

Thus, late-stage AD is better conceptualized as a heterogeneous and context-dependent redistribution of microglial functional programs under sustained pathological stress, rather than as a uniform progression from protective DAM to a fixed neurotoxic state (Figure 2).

### 3.2 Microglial remodeling along the tau pathological continuum

Microglia can exert bidirectional effects during the development and propagation of tau pathology. Experimental studies indicate that microglia can facilitate the spread of pathological tau through mechanisms including exosome-mediated transfer (Asai et al., 2015). In human AD, imaging studies have further shown that microglial activation is spatially associated with tau accumulation and progression across Braak stages (Pascoal et al., 2021). Together, these findings indicate that the relationship between microglia and tau pathology is functionally heterogeneous and dependent on pathological context.

Microglial responses to tau pathology are also transcriptionally heterogeneous and can be further shaped by the interaction between Aβ and tau. Experimental studies have identified disease-stage-associated microglial transcriptional signatures under combined Aβ and tau pathology (Kim et al., 2022), while alterations in microglial lipid homeostasis and autophagy can modify tau pathology and spreading in experimental models (X et al., 2021). Importantly, these transcriptional patterns should not necessarily be interpreted as stable tau-associated microglial subtypes. Rather, they may reflect differential engagement of inflammatory, lipid-metabolic, phagocytic, and stress-responsive programs according to pathological burden and local microenvironment. Recent human data further suggest that microglial state remodeling around the Aβ–tau pathological inflection point is associated with divergent trajectories toward dementia or resilience (Lu et al., 2026).

Thus, microglial involvement in tau pathology is better understood as a context-dependent balance among partially overlapping functional programs rather than as the sequential emergence of discrete tau-associated subtypes. Defining which of these responses promote tau clearance, propagation, or neurodegeneration—and under which pathological conditions—will be important for the development of microglia-directed therapeutic strategies (Shi et al., 2019; Leyns and Holtzman, 2017).

### 3.3 Regional and sex-dependent contexts of microglial remodeling

A substantial body of literature suggests that microglial responses differ across brain regions, including the hippocampus and cortex. Regional heterogeneity is also evident at the regulatory level. In the cerebral cortex, one study identified AD-associated cis-regulatory elements and candidate transcriptional regulators, linking altered regulatory architecture to disease-associated transcriptional changes in microglia (Anderson et al., 2023).

Hippocampal microglia exhibit sex differences during aging, which may be associated with the pathological progression of Alzheimer’s disease. Hippocampal microglia in aged female mice are more inclined to display disease-associated and aging-related phenotypes, suggesting that sex-related differences in microglial aging may contribute to differential vulnerability to AD-related pathology (Ocanas et al., 2023).

Taken together, regional differences in microglial profiles are better interpreted as spatially conditioned patterns of state engagement rather than as evidence of fixed region-specific subtypes. Anatomical location, local pathology, sex, and cellular microenvironment can jointly influence the relative prominence and functional consequences of disease-responsive microglial programs. Spatially resolved approaches are therefore required to determine how regional and neighborhood contexts shape microglial remodeling during AD progression.

### 3.4 Spatial transcriptomics and proteomics support a continuous, neighborhood-dependent model of microglial states

Recent spatial transcriptomic and proteomic studies add an important dimension to single-cell and single-nucleus analyses by preserving information about the anatomical and cellular neighborhoods in which disease-responsive microglial programs are expressed. Human spatial proteomic analyses have revealed substantial regional and microenvironmental variation in AD-associated microglial states, including variation in HLA-DR expression (Mrdjen et al., 2025). In experimental amyloidosis, spatially defined microglial states are also associated with distinct chromatin-accessibility profiles, indicating that local pathological environments can be linked to differences in both transcriptional and regulatory programs (Ardura-Fabregat et al., 2025). Moreover, spatial analyses of immune–microglial interactions show that proximity to local immune cells, including CD8^+^ T cells, can influence the relative prominence of MHC-II- and interferon-responsive microglial features (Kedia et al., 2024).

Collectively, spatially resolved studies argue against interpreting microglial clusters as spatially uniform or fixed cell populations. Instead, disease-responsive programs appear to vary along local pathological gradients and across distinct cellular neighborhoods, with plaque proximity, Aβ–tau burden, immune-cell interactions, and regional context shaping the relative engagement of overlapping transcriptional programs. Spatial heterogeneity should therefore be viewed as a continuous and neighborhood-dependent dimension of microglial plasticity rather than merely as regional differences in the abundance of discrete clusters.

## 4 Core regulatory mechanisms shaping microglial state remodeling

### 4.1 Regulatory roles of core risk genes

TREM2-DAP12/SYK signaling: TREM2 is better understood as a context-dependent signaling hub rather than a linear switch for beneficial DAM activation. TREM2 can recognize diverse lipid- and lipoprotein-associated ligands, and the nature and avidity of receptor–ligand interactions can influence receptor engagement and downstream responses (Wang et al., 2015; Yeh et al., 2016). Receptor organization is another important determinant of signaling strength: experimental enhancement of TREM2 clustering increases downstream SYK activation and can alter microglial metabolic and transcriptional responses (van Lengerich et al., 2023). AD-associated variants such as R47H may impair specific aspects of ligand recognition or downstream signaling, further illustrating that TREM2 activity cannot be reduced to a single uniform output (Wang et al., 2022; Yeh et al., 2016).

Downstream TREM2 signaling is also branched rather than strictly linear. In Aβ-associated microglial responses, TREM2 can engage both SYK-dependent and SYK-independent/DAP10-associated signaling routes, indicating that pathway selection can generate distinct functional outputs (Wang et al., 2022). TREM2 signaling is additionally coupled to microglial metabolic fitness: impaired TREM2 function disrupts mTOR-associated energetic and biosynthetic programs and compromises plaque-associated microglial responses (Ulland et al., 2017). Importantly, the consequences of TREM2 activity also vary with pathological stage. Experimental manipulation of TREM2 in amyloid models has produced different effects during early amyloid seeding and subsequent plaque-growth phases, indicating that increasing TREM2 signaling does not necessarily produce the same DAM-associated or pathological outcome throughout disease progression (Zhao et al., 2022). Thus, the biological consequences of TREM2 signaling reflect the combined influence of ligand identity, receptor clustering, downstream pathway engagement, microglial metabolic state, and disease stage rather than a uniform transition toward a beneficial DAM phenotype.

APOE genotype: APOE is another major determinant of microglial state remodeling in AD, particularly through its effects on lipid handling, inflammatory signaling, and interactions with TREM2-related pathways. APOE4 has been associated with altered microglial transcriptional responses, morphology, and lipid metabolism in AD, but these effects are heterogeneous and should not be interpreted as a uniform transition toward a single phagocytic or pro-inflammatory state (Haney et al., 2024; Serrano-Pozo et al., 2021; Kloske et al., 2023). Interactions between APOE and TREM2 may further modify these responses. Structural and computational studies have suggested that the combination of TREM2 R47H and APOE4 may alter interfacial flexibility, although the downstream functional consequences *in vivo* remain to be fully established (Lietzke et al., 2025). Taken together, APOE and TREM2 are better viewed as interacting regulators that shape microglial state remodeling through lipid-sensing, signaling, and metabolic mechanisms in a context-dependent manner, rather than as a pathway that uniformly promotes the formation of a single DAM state.

### 4.2 Immunometabolic reprogramming in microglial state remodeling

Microglial immunometabolic remodeling in AD extends beyond changes in glycolysis and lipid storage and involves coordinated alterations in mitochondrial function, cholesterol trafficking, fatty acid oxidation, glutamine utilization, and NAD^+^-dependent signaling. These metabolic processes are highly interconnected and can influence microglial energetic fitness, phagocytic competence, inflammatory signaling, and cellular plasticity. Importantly, their relative contributions are likely to vary according to pathological burden, genetic background, local microenvironment, and disease stage. To avoid conflating evidence across model systems, the discussion below explicitly distinguishes findings obtained in AD-relevant models from mechanistic observations derived from general inflammatory or toxicological paradigms and from hypotheses that remain to be validated in human AD.

#### 4.2.1 Glycolytic reprogramming and mitochondrial remodeling

Microglial activation is closely coupled to metabolic remodeling; however, current evidence does not support a universal or unidirectional switch from oxidative phosphorylation to glycolysis across all microglial states in AD. Experimental studies using CdTe/ZnS quantum dots or lipopolysaccharide (LPS) have shown that inflammatory activation can be accompanied by increased glycolysis and altered mitochondrial morphology, including enhanced mitochondrial fission (Wu et al., 2020; Nair et al., 2019). However, these findings were obtained primarily from toxicological or general inflammatory models rather than AD-specific systems and therefore should be interpreted as mechanistic evidence of microglial metabolic plasticity rather than as direct evidence for a uniform metabolic trajectory in AD (Lauro and Limatola, 2020).

AD-relevant studies instead indicate that preservation of mitochondrial energetic capacity and metabolic flexibility is critical for disease-responsive microglial functions. TREM2-deficient microglia exhibit defective mTOR signaling, reduced ATP availability, abnormal autophagic responses, and impaired biosynthetic fitness in the context of amyloid pathology, indicating that TREM2 contributes to the maintenance of microglial metabolic competence (Ulland et al., 2017). Conversely, TREM2 activation can enhance mitochondrial respiration, glucose oxidation, and metabolic reserve in human induced pluripotent stem cell-derived microglia and AD mouse models (van Lengerich et al., 2023). These observations suggest that mitochondrial remodeling in AD involves not only changes in substrate preference but also alterations in respiratory capacity, mitochondrial dynamics, and quality-control pathways such as mitophagy. Thus, rather than representing a fixed transition from oxidative phosphorylation to glycolysis, microglial metabolism is better viewed as a context-dependent redistribution of energetic pathways shaped by pathological burden, inflammatory signaling, TREM2 activity, and mitochondrial integrity.

#### 4.2.2 Cholesterol handling, lipid-droplet homeostasis, and fatty acid oxidation

Lipid dysregulation represents another major component of microglial immunometabolic remodeling in AD. Microglia are continuously exposed to lipid-rich material derived from myelin, neuronal membranes, and other phagocytosed cellular debris. Persistent uptake can exceed the capacity of lysosomal processing and lipid export, favoring intracellular lipid-droplet accumulation. Lipid-droplet-associated microglial states have been identified in aging and AD-related contexts and are associated with impaired phagocytic competence and increased inflammatory signaling (Haney et al., 2024; Marschallinger et al., 2020; Claes et al., 2021). Importantly, however, lipid accumulation should not be regarded simply as a consequence of excessive lipid uptake; it also reflects abnormalities in intracellular lipid processing, cholesterol trafficking, and mitochondrial lipid utilization.

One representative consequence of persistent metabolic and phagocytic stress is the self-reinforcing interaction among impaired lipid handling, reduced phagocytic competence, and inflammatory activation, as schematically illustrated in Figure 3. This feedback loop represents one component of the broader immunometabolic network operating in AD microglia.

**FIGURE 3:**
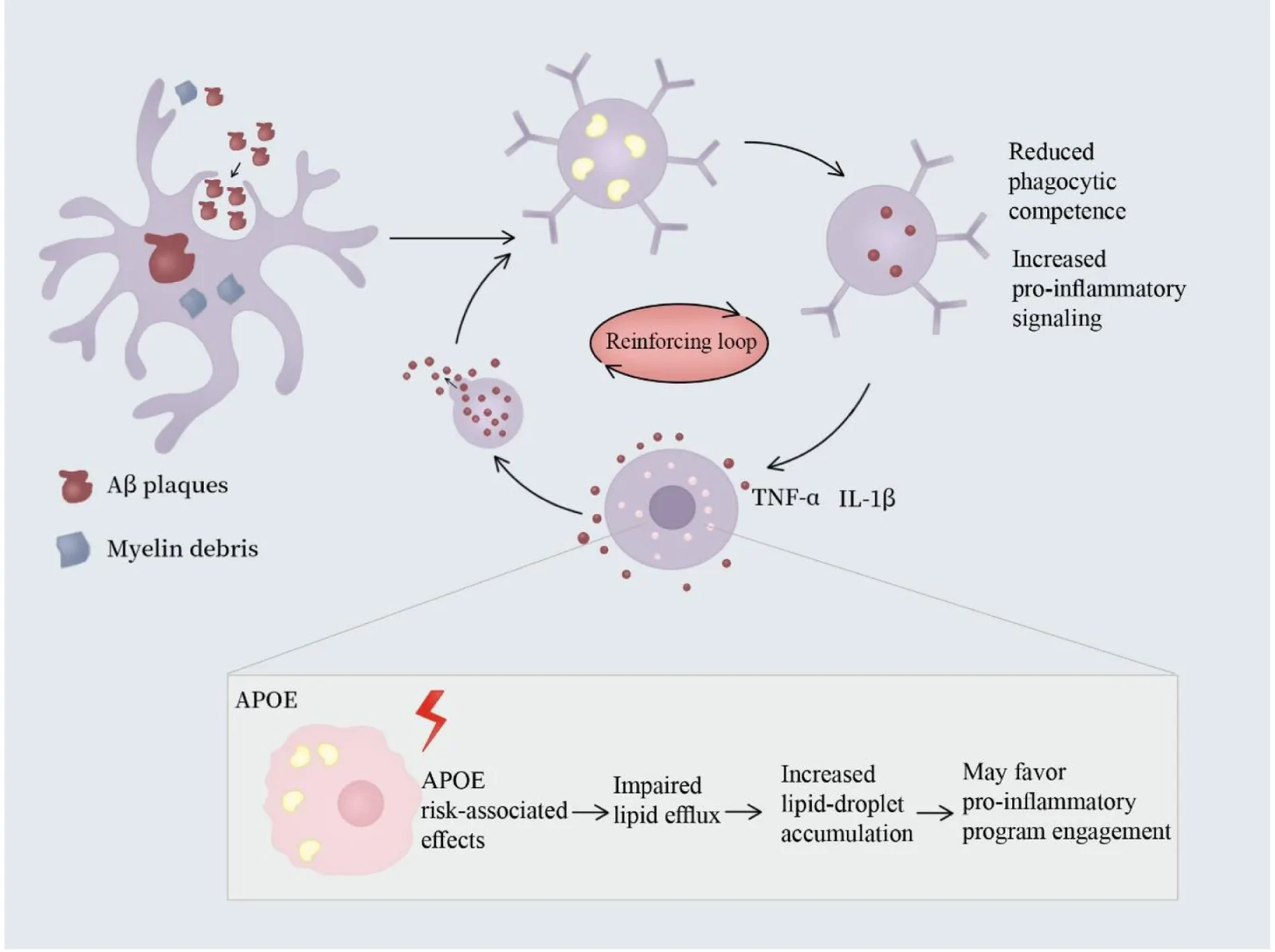
Conceptual Model of Interacting Phagocytic Dysfunction and Inflammatory Signaling in AD Microglia. Pathological inputs: In the Alzheimer’s disease brain, microglia are continuously exposed to pathological stimuli, including Aβ plaques and myelin debris. Persistent uptake of these materials can impose substantial metabolic and phagocytic stress on microglia. Reinforcing feedback: Sustained pathological and phagocytic stress can contribute to reduced phagocytic competence and increased production of pro-inflammatory mediators, including TNF-α and IL-1β. These processes can mutually reinforce one another, forming a reinforcing feedback loop rather than an obligatory progression toward a fixed dysfunctional state. APOE-related modulation: APOE risk-associated effects may further exacerbate defects in lipid handling and favor greater engagement of pro-inflammatory programs, depending on the surrounding pathological and metabolic context. Overall, the schematic illustrates a context-dependent reinforcing interaction among impaired lipid handling, reduced phagocytic competence, and inflammatory signaling rather than an obligatory transition toward a fixed pro-inflammatory microglial state.

Cholesterol metabolism is particularly relevant to this process. Under chronic phagocytic challenge, TREM2-deficient microglia can internalize myelin-derived material but show impaired clearance of cholesterol, resulting in accumulation of cholesteryl esters. Similar abnormalities have been observed in human iPSC-derived microglia, supporting a role for TREM2 in coordinating cholesterol transport and metabolic adaptation (Nugent et al., 2020). More recent metabolic and lipidomic analyses in APP-transgenic mice further indicate that higher TREM2 expression is associated with increased oxidative phosphorylation, improved cholesterol homeostasis, and greater metabolic capacity (Feiten et al., 2026). These observations connect AD genetic risk pathways directly with microglial cholesterol handling.

Fatty acid oxidation (FAO) provides an additional link between lipid storage and mitochondrial energy production. TREM2 activation in human iPSC-derived microglia reduces triglyceride-rich lipid droplets, increases acyl-carnitine production, and enhances CPT1-dependent mitochondrial FAO and respiratory capacity (van Lengerich et al., 2023). Therefore, the accumulation of lipid droplets in dysfunctional microglia may reflect an imbalance among lipid uptake, lysosomal processing, cholesterol storage and efflux, and mitochondrial fatty acid utilization rather than simply increased lipid burden. APOE and TREM2 signaling are likely to influence several points within this metabolic network, thereby shaping the functional consequences of lipid-associated microglial states.

#### 4.2.3 Glutamine metabolism and NAD^+^-dependent signaling

Beyond glucose and lipid metabolism, glutamine utilization has emerged as another metabolic pathway capable of regulating microglial inflammatory activity. Glutamine provides carbon and nitrogen for tricarboxylic acid-cycle anaplerosis, amino-acid synthesis, and other biosynthetic processes. In APP/PS1 mice and activated microglial cultures, increased glutamine metabolism accompanies NLRP3 inflammasome activation. Pharmacological inhibition of glutaminase decreases glutamine-derived anaplerotic flux, suppresses mTORC1 signaling through AMPK activation, enhances mitophagy, reduces intracellular reactive oxygen species, and ultimately attenuates NLRP3 inflammasome activation (Zhang et al., 2024). These findings provide an AD-relevant mechanistic link between nutrient utilization, mitochondrial quality control, and inflammatory signaling.

Nicotinamide adenine dinucleotide (NAD^+^) metabolism represents another important interface between cellular energetics and immune regulation. In APP/PS1 mice, reduced NAD^+^ availability is associated with neuroinflammation, whereas supplementation with the NAD^+^ precursor nicotinamide riboside increases brain NAD^+^ levels, promotes mitophagy, and reduces microglial activation, NLRP3-associated inflammation, and cGAS–STING signaling (Hou et al., 2021). NAD^+^ also serves as an essential substrate for sirtuin deacetylases. In aging and tauopathy models, loss of microglial SIRT1 enhances IL-1β expression and contributes to cognitive impairment, providing additional evidence that NAD^+^-dependent signaling can influence inflammatory microglial programs (Cho et al., 2015). Nevertheless, most evidence linking NAD^+^ metabolism to specific microglial states remains preclinical, and the extent to which these pathways define microglial responses in human AD requires further investigation.

Collectively, these findings indicate that microglial immunometabolism in AD cannot be adequately described by a simple oxidative phosphorylation-to-glycolysis switch or by lipid-droplet accumulation alone. Instead, mitochondrial remodeling, cholesterol trafficking, fatty acid oxidation, glutamine utilization, and NAD^+^-dependent signaling form an interconnected metabolic network that influences microglial energetic fitness, phagocytic competence, inflammatory signaling, and cellular plasticity. The relative engagement of these pathways is likely to vary according to disease stage, pathological burden, genetic background, and local microenvironment, further supporting a dynamic rather than fixed model of microglial state regulation.

## 5 Therapeutic implications and future perspectives

### 5.1 Potential microglia-directed therapeutic strategies

Based on current evidence, microglia-directed immunotherapy is better framed as context- and state-aware modulation rather than selective activation or suppression of fixed cellular subtypes. TREM2 agonism is being investigated as a strategy to modify plaque-associated microglial responses; however, its effects are likely to depend on receptor engagement, downstream pathway configuration, microglial metabolic fitness, and disease stage rather than uniformly enhancing a beneficial DAM phenotype (Wang et al., 2022; Ulland et al., 2017; van Lengerich et al., 2023; Zhao et al., 2022). Similarly, interferon-responsive programs may represent potential therapeutic targets when signaling becomes persistent or excessive, but their functional consequences are context dependent (Roy et al., 2022; Roy et al., 2020). The timing of intervention is also likely to be important, because manipulation of TREM2 signaling can produce different outcomes across phases of amyloid pathology, and the optimal therapeutic window remains uncertain (Zhao et al., 2022). In more advanced pathological contexts, interventions targeting maladaptive inflammatory pathways, including inflammasome, chemokine, or complement signaling, may be considered, although broad suppression of microglial activity could also interfere with beneficial homeostatic or protective functions. Finally, pathway-directed strategies should account for signaling branch points rather than assuming a single linear TREM2–DAP12–SYK axis. Although SYK represents an important downstream component of TREM2-DAP12 signaling, TREM2-mediated responses are not exclusively SYK dependent (Wang et al., 2022). Thus, future microglia-directed therapies will likely require biomarker-informed, stage- and state-aware approaches rather than a simple paradigm of broadly enhancing microglial activity early and suppressing it later.

However, substantial translational barriers remain. Because DAM states are partially overlapping and context dependent rather than discrete therapeutic targets, selectively suppressing a maladaptive program while preserving homeostatic, plaque-containment, and tissue-supportive functions remains challenging (Fumagalli et al., 2025; Paolicelli et al., 2022; Sun et al., 2023; Mancuso et al., 2024). This concern is particularly relevant to broad modulation of interferon, inflammasome, complement, or SYK-associated signaling, because the functional consequences of these pathways can vary with pathological context and disease stage. Therapeutic windows are likewise uncertain, as illustrated by the stage-dependent consequences of TREM2 manipulation in amyloid models (Zhao et al., 2022). Central nervous system delivery represents an additional practical limitation, particularly for biologic therapies; notably, recent TREM2 agonist approaches have incorporated a blood–brain barrier transport vehicle to enhance brain exposure (van Lengerich et al., 2023). Finally, most currently defined microglial states are identified through tissue-based single-cell, single-nucleus, or spatial profiling rather than through validated *in vivo* biomarkers (Fumagalli et al., 2025; Paolicelli et al., 2022; Sun et al., 2023; Green et al., 2024; Mathys et al., 2024). Consequently, identifying patients with a particular disease-stage-specific microglial state, selecting the appropriate intervention, and monitoring target engagement in living patients remain major unresolved challenges. These limitations suggest that TREM2 agonism, interferon or SYK pathway modulation, inflammasome or complement inhibition, and state-directed interventions should currently be regarded as mechanistically informed experimental strategies rather than established precision therapies.

### 5.2 Current challenges and future directions

Despite the conceptual shift from a simple dichotomy to a complex, dynamic functional spectrum of microglia, translation into clinical strategies remains challenging. First, spatiotemporal integration of spatial transcriptomics, spatial proteomics, and single-cell sequencing data is needed to map microglial state distributions, neighborhood-dependent transcriptional programs, and their relationships with local pathology, alongside higher-resolution *in vivo* imaging to capture dynamic state remodeling and cell–cell interactions. Second, species differences preclude direct extrapolation from mouse models to humans, and region- and sex-driven microglial heterogeneity remains incompletely characterized. Spatial transcriptomics offers a critical means to link microglial states and transcriptional programs to specific anatomical sites and neuropathological structures, while combining cell-cell communication analyses with advanced tracing technologies can decode the complex signaling between microglia, astrocytes, and neurons within the brain microenvironment. Future paradigms should integrate advanced model systems—such as 3D brain organoids and humanized chimeric models—to validate microglial states in human-relevant settings, providing platforms for mechanistic validation, biomarker development, and evaluation of candidate microglia-directed interventions.

### 5.3 Controversies and unresolved questions

Despite rapid advances in single-cell and spatial profiling, several fundamental questions regarding microglial heterogeneity in AD remain unresolved. First, the biological status of many transcriptomically defined populations remains uncertain. Clusters identified by single-cell or single-nucleus analyses do not necessarily correspond to stable biological subtypes, because their boundaries can vary with clustering resolution, annotation strategy, tissue source, pathological context, and analytical methodology (Fumagalli et al., 2025; Paolicelli et al., 2022; Sun et al., 2023; Lu et al., 2026; Mrdjen et al., 2025; Green et al., 2024; Miyoshi et al., 2024; Kosoy et al., 2025; Mathys et al., 2024). A major challenge is therefore to determine which reported profiles represent transient transcriptional programs, which correspond to more persistent functional states, and whether any satisfy the criteria for stable biological subtypes. Cross-dataset harmonization, spatial validation, and functional characterization will be required before nomenclature can be reliably linked to biological identity.

Second, the trajectories connecting microglial states remain incompletely resolved. Most human AD datasets are cross-sectional and cannot establish whether individual microglia move sequentially between the states inferred from transcriptomic analyses. Human *postmortem* studies are additionally influenced by disease-stage heterogeneity, brain region, genetic background, agonal and *postmortem* factors, whereas experimental mouse models reproduce only selected components of human AD pathology (Fumagalli et al., 2025; Gosselin et al., 2017; Sun et al., 2023; Mancuso et al., 2024; Green et al., 2024; Miyoshi et al., 2024; Kosoy et al., 2025; Mathys et al., 2024). Consequently, apparent early-, intermediate-, and advanced-stage patterns should be interpreted as recurrent population-level tendencies rather than direct evidence that individual microglia follow a universal lineage trajectory.

Third, the functional consequences of major regulatory and metabolic pathways remain strongly context dependent. TREM2 signaling can support plaque-associated responses and metabolic fitness, yet its consequences vary with ligand context, downstream pathway engagement, and pathological stage (Wang et al., 2022; Ulland et al., 2017; van Lengerich et al., 2023; Zhao et al., 2022). Likewise, metabolic remodeling cannot currently be reduced to a universal oxidative phosphorylation-to-glycolysis switch: some mechanistic evidence derives from general inflammatory or toxicological models, whereas AD-related studies support broader alterations in mitochondrial function, lipid handling, fatty acid oxidation, glutamine metabolism, and NAD^+^-dependent signaling (Ulland et al., 2017; van Lengerich et al., 2023; Haney et al., 2024; Marschallinger et al., 2020; Wu et al., 2020; Nair et al., 2019; Lauro and Limatola, 2020; Claes et al., 2021; Zhang et al., 2024; Hou et al., 2021; Cho et al., 2015). An important unresolved question is whether these metabolic changes actively drive particular microglial states or instead arise partly as consequences of pathological stress and altered cellular function.

Finally, a central unresolved translational question is whether molecularly defined microglial programs can be measured reproducibly in patients, linked prospectively to disease progression or treatment response, and selectively modulated without disrupting beneficial homeostatic or tissue-protective functions. Addressing this question will be essential for translating the rapidly expanding molecular taxonomy of microglia into biologically and clinically meaningful therapeutic frameworks.

## 6 Conclusion

Research on microglia has progressed beyond the classical M1/M2 dichotomy toward a dynamic and multidimensional view of microglial heterogeneity in AD. Rather than constituting a collection of fixed biological subtypes, recurrent DAM-associated, interferon-responsive, inflammatory, proliferative, antigen-presentation-associated, and metabolic programs may coexist, overlap, and change in relative prominence according to pathological burden, anatomical location, local microenvironment, genetic background, metabolic status, and disease stage. This review integrates these disease-responsive programs with Aβ and tau pathology to highlight how their context-dependent remodeling shapes microglial functions across the spatiotemporal course of AD.

Future research should integrate single-cell and spatial multi-omics, longitudinal clinical data, and human-relevant model systems to distinguish transient transcriptional programs from more persistent functional states and to determine which microglial responses are protective, maladaptive, or context dependent. Defining these relationships will be essential for developing stage- and state-aware therapeutic strategies that preserve beneficial microglial functions while limiting maladaptive inflammatory and metabolic responses.
